# A study on population dynamics in “Belt & Road” countries and their implications

**DOI:** 10.1007/s42379-018-0007-y

**Published:** 2018-06-25

**Authors:** Mengni Chen, Paul S. F. Yip

**Affiliations:** 1 Department of Social Work and Social Administration, The University of Hong Kong, Pokfulam, Hong Kong; 2 Wittgenstein Centre (IIASA, VID/ÖAW, WU), Vienna University of Economics and Business (WU), Welthandelsplatz, 2, Level 2, 1020 Vienna, Austria

**Keywords:** Belt and road initiative, Population dynamics, Demographic heterogeneity, Economic development, Well-being

## Abstract

China’s Belt & Road (“B&R”) initiative has attracted much attention in recent years. Many studies have assessed the potential impacts of this initiative from the economic, political, foreign relations, and military perspectives. However, very little attention has been paid to examine the opportunities and challenges of this new initiative from a demographic perspective. Therefore, this study investigates into the population dynamics of 65 countries in the B&R region, and explores the demographic dynamics of this regional cooperation model. Based on graphic visualization, the demographic heterogeneities within the B&R region, in terms of current stages of demographic transitions, population age structure, and demographic windows of opportunities have been revealed. Furthermore, a model-based cluster analysis has been conducted to classify the B&R countries into two groups. One group consists of countries with younger population, low education access, and larger gender gap in the labor market, while the other group comprises of more aged population and has better performances in economic development, education enrolment, and female labor participation. The demographic windows of opportunities in the former group will remain open for the coming decades; whereas the windows have already closed or is about to close very soon in the latter group. The former group should take lessons from the latter concerning how to better prepare themselves for the demographic transition. Moreover, the curvilinear relationship between wealth and well-being has a complicated structure and the B&R initiative should focus not only on economic development but also on the human well-being.

## Introduction

In September 2013, during his visit to Kazakhstan, President Xi of China put forward building up “the Silk Road Economic Belt” as a new regional cooperation development model, later in the same year in October, he again called for “the twenty-first Century Maritime Silk Road” in a speech to the Indonesian parliament (Swaine 2015). The two proposals have now been officially named as the “Belt and Road Initiative” (hereinafter Belt and Road abbreviated as “B&R”). This B&R initiative is to break geographic limitations and strengthen an inclusive, balanced, and open relationship, covering five cooperation priorities- policy coordination, facilities connectivity, trade facilitation, financial cooperation, and people-to-people bonds (Huang 2016; Johnston 2016).

Currently, China is facing four big challenges which motivated the government to in time adopt a new development model. First, with the continuous increase in labor costs over the past decades, now China has been losing its advantage and competitiveness in labor-intensive industries (Li et al. 2012). Second, export, the main pillar of China’s economy, has recently slowed down, especially after the global financial crisis in 2008 (Ma and Van Assche 2009) and the potential trade war with the USA. Third, the land-intensive and resource-intensive economic growth model has caused extensive environmental problems, which have now become a serious concern to the sustainable development of the country (Chunmei and Zhaolan 2010; Fang et al. 2009). Fourth, the rapid population aging and the shrinkage of labor force in the coming decades are likely to slow down China’s future economic growth (Chen et al. 2018; Johnston 2016; Minghao 2016). Therefore, the B&R strategy is initiated, with aims to stimulate socioeconomic development not only within the country but also across countries in a vast region, to search for mutual benefits and to improve people’s livelihoods, employment, and the environment (Huang 2016; Liu and Dunford 2016).

At present, the B&R countries are a home to 4.4 billion people, covering 64% of the world’s population and 65 countries across Asia, Europe, and Africa; this region generates GDP of US$21 trillion, about 30% of the global GDP (Swaine 2015; Zhai 2018). Whether the B&R initiative can be a mutually beneficial strategy in the future depends on various factors (including political, economic, social, cultural factors, etc.) and how those factors interplay.

The population dynamics in the B&R region are one of the essential factors which would determine the pace of economic prosperity and social development. However, this factor has not received the deserved attention. Some member countries have large shares of old population, whereas other countries have high proportions of young population (Johnston 2016). This indicates that with well utilization, the demographic heterogeneities among the B&R countries might lay the foundation for economic complementarity and win–win outcomes. Therefore, a good understanding of the population profiles in China and other member countries will aid long-term strategic planning of the B&R initiative. Hence, this research conducts a comprehensive investigation into this crucial factor, to examine (1) in what demographic dimensions the heterogeneities exist; (2) the potential challenges and opportunities; (3) how this initiative can benefit all the countries as well as the people living in the B&R region.

## Data and methods

The data required in this study include crude birth rate (“CBR”), crude death rate (“CDR”), proportions of the elder population (i.e. those aged 65 and above), total fertility rate (“TFR”), natural growth rate, life expectancy (“LE”), total dependency ratio, labor force participation rate by gender, tertiary school enrollment, GDP per capita (constant 2010 USD), employment by industry, proportional contribution to GDP by industry, and the happiness index. The sources of the data are listed in Table 1. The methods adopted in this study include graphic visualization and model-based clustering. These analyses are performed in software R.

**Table 1:** The sources of the data required for the descriptive analyses

Data	Period	Source
Crude birth rate (“CBR”)	2014	World Bank
Crude death rate (“CDR”)	2014	World Bank
Proportions of the elderly population	1960–2050	World Bank
Total fertility rate (“TFR”)	2010–2014	World Bank
Natural growth rate (“NGR”)	2010–2014	World Bank
Life expectancy (“LE”)	2010–2014	World Bank
Total dependency ratio	1960–2050	World Bank
Labor force participation rate by gender	2010–2014	World Bank
Tertiary school enrollment by gender	2010–2014	World Bank
GDP per capita (constant 2010 USD)	2010–2014	World Bank
Employment by industry (%)	2010–2014	World Bank
Added value to GDP by industry (%)	2010–2014	World Banky
Happiness Index	2015	World Happiness Report

First, different stages of demographic transitions among the B&R countries, in terms of crude birth and death rates, are presented. Second, the age structures, in terms of proportions of the elderly population, are visualized. Third, a cluster analysis based on three factors namely, the total fertility rate, life expectancy, and natural population growth rate is conducted. Then, for each cluster, the socioeconomic characteristics, employment and industrial structures, demographic windows of opportunities, and human well-being are demonstrated.

## Results

### Stages of the demographic transitions in the B&R region

**Fig. 1:**
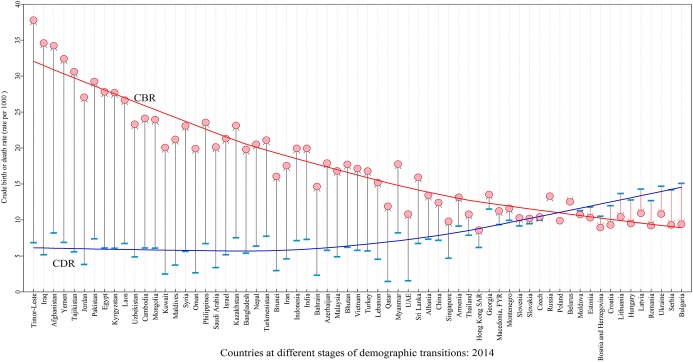
The current stages of demographic transitions in 65 B&R countries

The whole demographic transition has been classified into five stages: in the first stage, both fertility and mortality are very high, leading to a stable or slow increase of population; in the second stage, the population grows very rapidly, as fertility remains at a high level while the mortality starts to fall greatly; in the third stage, the population growth slows down, with fertility falling and mortality falling more slowly; in the fourth stage, the population size stabilizes again, with fertility and mortality remaining at very low level; and in the fifth stage, fertility continues to decline to a level even lower than mortality, therefore the population size starts to decrease (Max and Esteban 2018). Figure 1 lists and visualizes the current stages of demographic transitions among the 65 B&R member countries, in terms of CBRs and CDRs. As shown, there are large variations in this region, ranging from the lowest natural growth rate in Bulgaria (− 5.6 per 1000 persons) to the highest in Timor-Leste (30.9 per 1000 persons). In comparison to the five stages of the demographic transition in Fig. 8 (in the “Appendix”), it can be seen that about 13 countries in the B&R region are in the fifth stage, experiencing depopulation, and most of them are in Eastern Europe; half of the B&R countries are in the fourth stage, experiencing slow increase of population; and about 20 countries located in South Asia, Middle Asia, and Africa are in the third stage, with natural growth rates of 15 or above per 1000 persons. This figure highlights the heterogeneities of population dynamics, in terms of the current stages of demographic transitions. In the B&R region, if appropriate training and education were available, the rich human resources in countries with growing and young populations could to be tapped and transformed into productive labor force, while other countries with depopulation may face the shrinkage of labor force in the coming decades. Especially in the Eastern European countries, if the fertility rates do not reverse and the emigration (to Nordic and western Europe) continues to grow, those countries may see “the steepest drops in the size of working-age population within Europe” in the future (Romei 2016).

### The trends of population aging in the B&R countries over the period 1960–2050

**Fig. 2:**
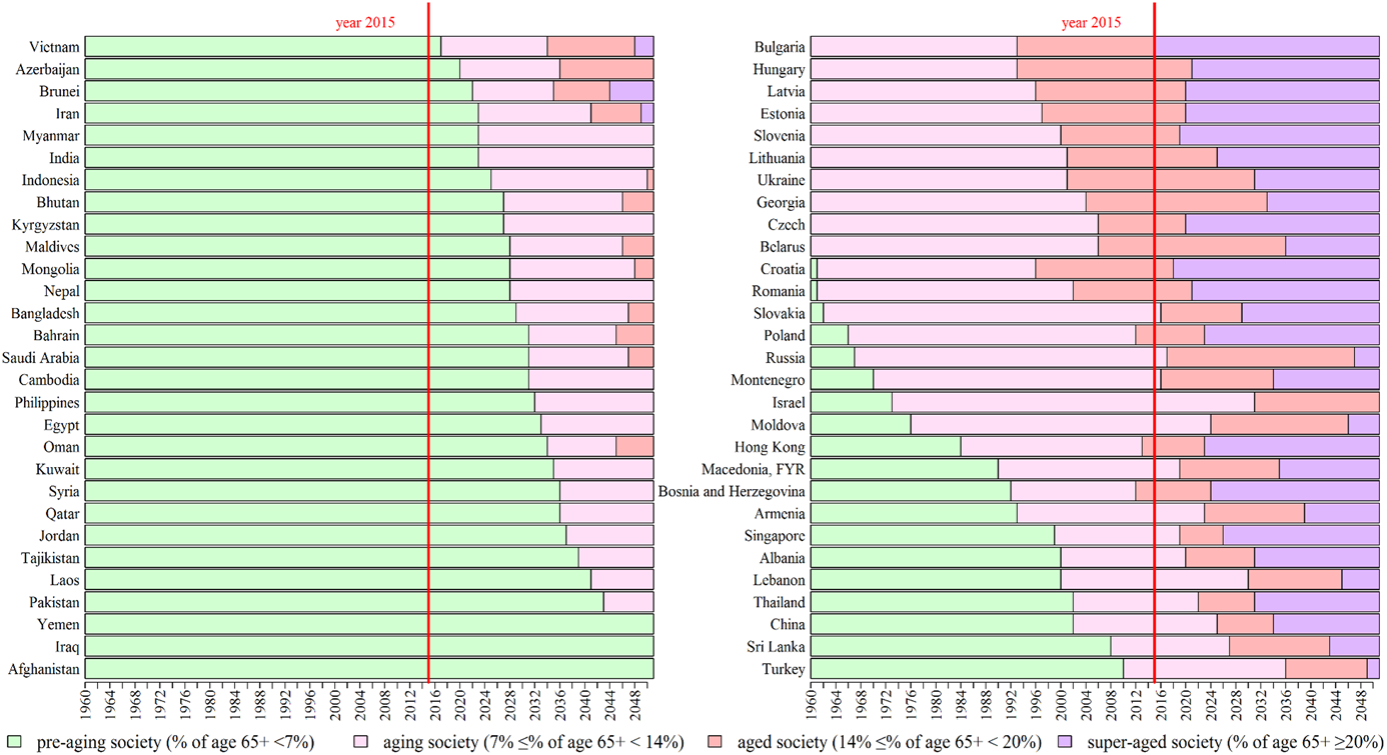
Population aging over the period 1960–2050. Due to limitation of data, seven countries (United Arab Emirates, Kazakhstan, Malaysia, Serbia, Turkmenistan, Timor-Leste, Uzbekistan) are not included

After a brief look at the pattern of population growth, the population age structure is explored, with focus on the population aging. According to the World Health Organization and the United Nations, the percentage of people aged 65 and above in the total population, is one of the conventional indicators, often used to measure the speed of population aging (UN 2002; WHO 2011). By international standards: if this percentage is under 7%, it is a pre-aging society; if the percentage ranges from 7 to 14%, it is classified as an aging society; if the percentage ranges from 14 to 21%, it is an aged society; and if the percentage is over 21%, it is categorized as a super-aged society (Tahara 2016; Yoshifumi 2016). As shown in Fig. 2, 29 countries (mostly in Middle and South Asia, Africa) in the B&R region are currently “pre-aging” societies, 14 of which will become “aging societies” in 2050, 9 of which will become “aged societies”, 3 of which (i.e. Vietnam, Azerbaijan, Brunei, and Iran) will turn into super-aged countries by the middle of the twenty-first century, and the rest (Yemen, Iraq, and Afghanistan) will still be pre-ageing societies from the present to 2050. Another 29 countries on the right section of Fig. 2 (located in Eastern Europe, East and Southeast Asia) are now either “aging” or “aged”, all of which will become super-aged by 2050. It seems that although about half of the B&R countries are currently with young populations, the rapid aging trends will sweep across the B&R region in the coming decades. This indicates that sooner or later, population aging will be a common challenge to all the B&R countries. However, at this moment, the diversity of population age structures in the B&R region still leaves many opportunities for regional cooperation, international migration, and exchange of human capital, knowledge, and technology, through which those pre-aging countries can speed up its socioeconomic development, and those aging societies can sustain stable economic growth.

### The demographic clusters and windows of opportunities among the B&R countries

**Fig. 3:**
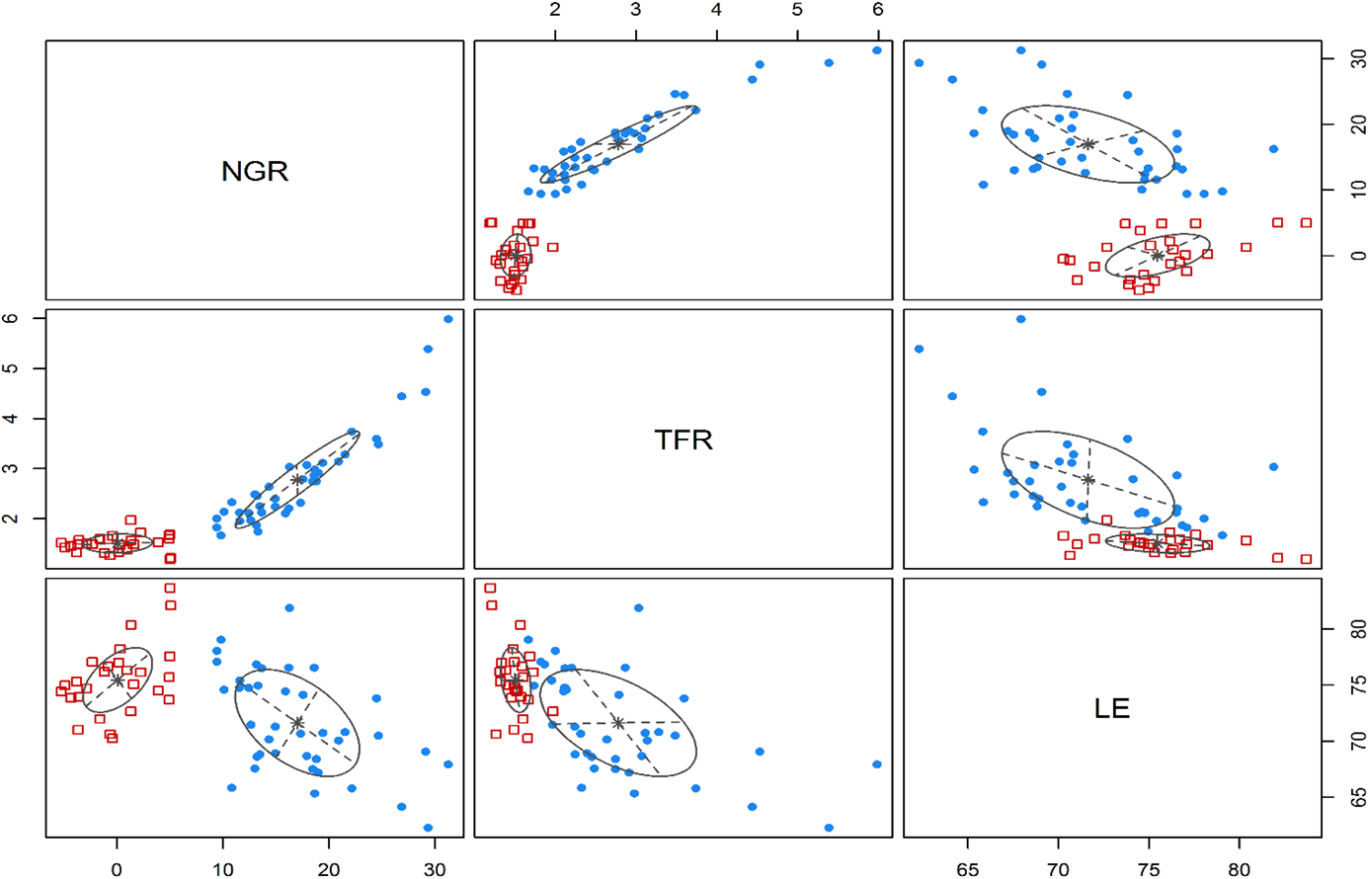
The demographic clusters in the B&R countries. “NGR” refers to the natural growth rate, “TFR” refers to the total fertility rate, and “LE” refers to the life expectancy; the cluster analysis is performed based on average levels of the three indicators during 2010–2014

**Fig. 4:**
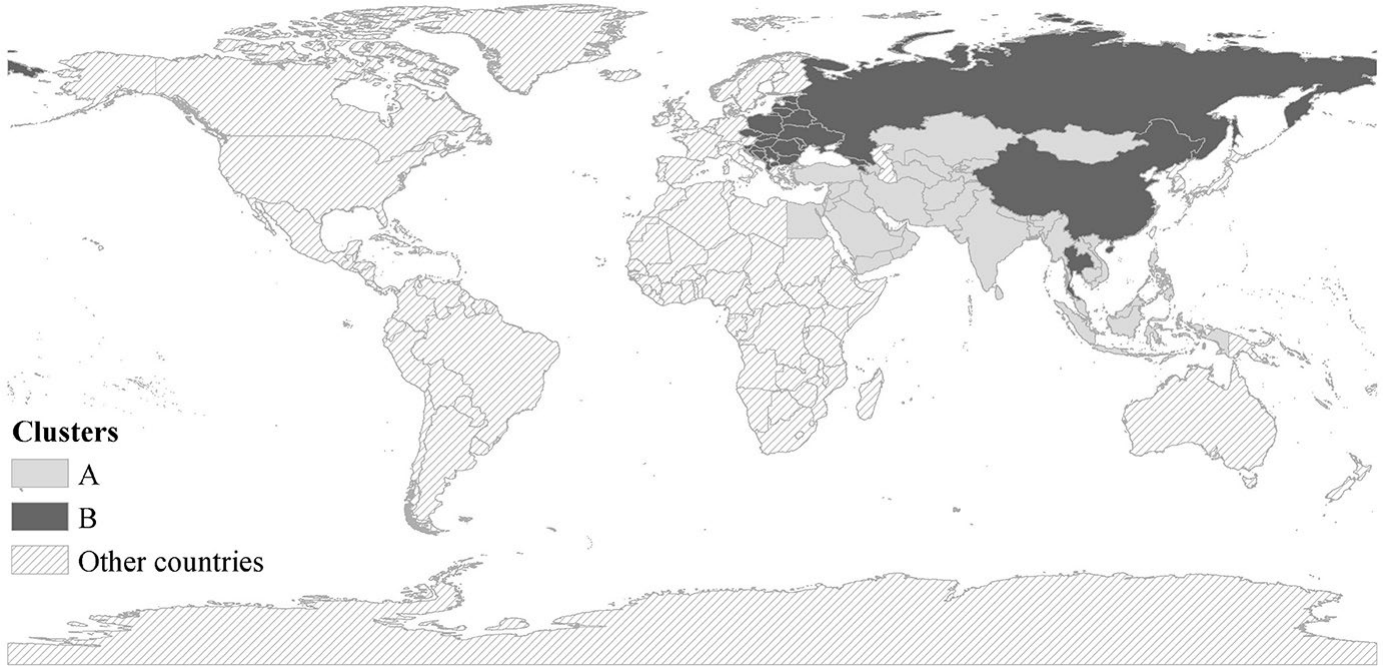
The geographic locations of the two clusters

Based on the natural population growth rates, total fertility rates, and life expectancies over the period 2010–2014, a model-based cluster analysis is conducted to classify the 65 B&R countries into two groups (see Fig. 3). Furthermore, Fig. 4 shows the geographic locations of the countries in the two clusters, and Table 2 summarizes their demographic and socioeconomic characteristics, as well as their employment and industrial structures. As shown, there are 39 countries in Cluster A, most of which are in Western, Middle and South Asia as well as Africa; 26 countries are in Cluster B, geographically located in East Asia and Eastern Europe. As Table 2 shows, Cluster A has higher natural population growth rate, higher fertility rate, and shorter life expectancy, whereas Cluster B has stabilized population size, sub-replacement fertility rate and longer life expectancy. Cluster A consists of countries with lower urbanization rate and lower GDP per capita (with average of 9932.57 USD); conversely, Cluster B consists of countries with higher proportion of urban population and higher GDP per (about 12,000 constant USD). It is noteworthy that Cluster B has much better performances in female labor force participation and tertiary school enrolment (among both males and females), and the gender gap in labor force participation is much smaller among Cluster B. It is also interesting to notice that Cluster A has higher male labor force participation than Cluster B. This, to some extent, can be explained by the male bread-winner model in those less developed countries where men are supposed to work outside the home to provide family income while women are supposed to stay at home for housework and childcare. Despite some cultural and religion factors, these findings still indicate that among the countries in Cluster A, there is much room for improvement in education and gender equity in the labor market.

**Table 2:** Socioeconomic profiles of the B&R countries in two clusters

Cluster	A	B
No. of countries	39	26
Demographic characteristics		
Natural population growth rates (per 1000 persons)	17.09	0.04
Total fertility rates (No. of children per women)	2.79	1.50
Average life expectancies (years)	71.57	75.55
Socioeconomic characteristics		
Percentage of urban population (% of total)	53.77%	63.17%
Male tertiary school enrollment (gross)	26.84%	51.47%
Female tertiary school enrollment (gross)	29.85%	69.79%
Male labor force participation rates	77.19%	65.88%
Female labor force participation rates	42.57%	50.36%
Average GDP constant 2010 USD	9932.57	11897.69
Employment structure		
Employment in agriculture (% in total employment)	27.88%	16.23%
Employment in industry (% in total employment)	22.81%	25.89%
Employment in service (% in total employment)	49.31%	57.88%
Industrial structure		
Agriculture added value (% of GDP)	12.93%	7.30%
Industry added value (% of GDP)	37.14%	29.24%
Service added value (% of GDP)	49.93%	63.46%

The values of the indicators are the average values over 2010–2014

The two clusters also differ from each other in the employment and industrial structures. As shown in Table 2, in cluster A, about 28% of workers are employed in the agriculture sector, contributing 13% to the GDP; about 23% work in the industry sector, contributing about 37% to the GDP; about 49% work in the service sector, contributing about 50% to the GDP. In contrast, in cluster B, only 7% of the GDP can be attributed to the agriculture sector which only accounts for 16% of the employment, while almost two-thirds of the GDP are contributed by the service sector, which absorbs 58% of the employment. On one hand, these structural differences reflect the different levels of industrialization and different stages of economic development in the two clusters of B&R countries. On the other hand, these heterogeneities can also be the foundations for economic complementarity and cooperation in the B&R region, by promoting trade, technology exchanges, and infrastructure connectedness.

**Fig. 5:**
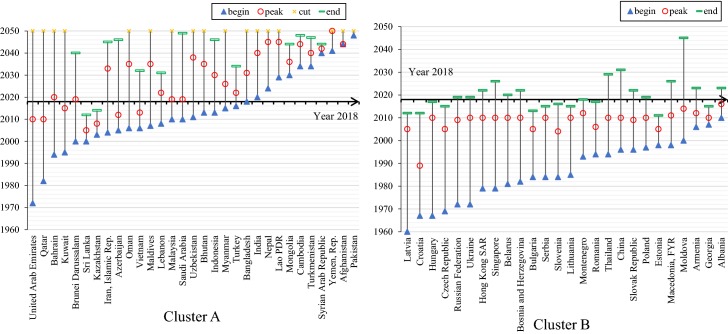
The demographic windows of opportunities in the B&R countries. In cluster A, the cut level means that the total dependency ratio in 2050 is still less than 0.5. There are 8 countries in Cluster A where the demographic window has not opened yet; these 8 countries are Egypt, Iraq, Israel, Jordan, Kyrgyz Republic, Philippines, Tajikistan, Timor-Leste

Moreover, the demographic windows of opportunities in the two clusters over the period of 1960–2050 have been visualized. Theoretically and historically, when the fall of mortality is followed by a rapid fertility decline, the proportion of working-age population will increase and the demographic window will appear; and when the proportion reaches its maximum level, the window will open to the largest extent. Therefore, the demographic window of opportunity can be seen as “a direct consequence of the demographic transition” (Vallin 2005). In existing literature, the technical definition for the demographic window may vary, depending on which indicator is referred. These indicators include the proportion of working-age population, the ratio of working-age to non-working age population, the child dependency ratio, the elderly dependency ratio, and the total dependency ratio (Bloom and Canning 2004; Peng 2005; Vallin 2005). Here, the total dependency ratio and the proportion of working-age population are adopted. When the total dependency ratio ranges from 0 to 0.5—meaning that more than 67% of population are in the working age, it is defined as a period when the demographic window is open (Peng 2005). This “golden age structure” will provide potential advantages for rapid economic growth (Peng 2005; Vallin 2005). And when the total dependency ratio is larger than 0.5, the window is closed. As shown in Fig. 5, there are great differences between the two clusters. In Cluster A, there are 8 countries where the windows of opportunities will not appear in 2018–2050 (see the Note); there are 11 countries (i.e. Bangladesh, India, Nepal, Laos, Mongolia, Cambodia, Turkmenistan, Syria, Yemen, Afghanistan, and Pakistan) where the windows will open one after another in the coming three decades; in other countries (except for Sri Lanka and Kazakhstan), the demographic window is currently open, providing favorable demographic conditions for socioeconomic development. The pattern of Cluster B however, is in big contrast to Cluster A. In the majority of those countries, the demographic window has opened before 2000; there are at present 11 countries where the windows have already closed; and in most of the rest 15 countries, the windows are about to close very soon.

**Fig. 6:**
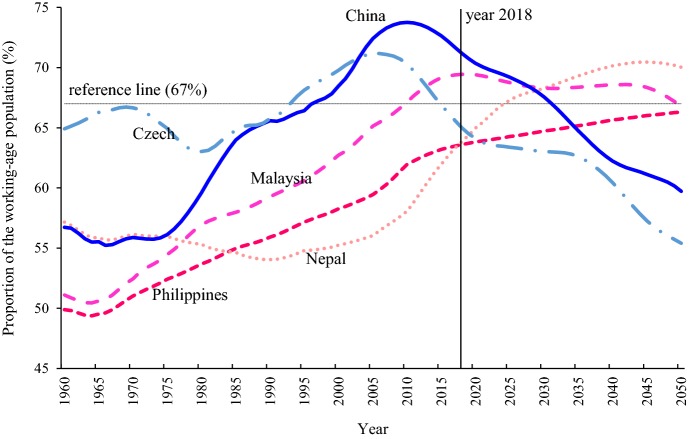
the proportion of working age population (age 15–64) over 1960–2050 in five selected countries. The reference line means that the proportion of working-age population is 67%, when the total dependency ratio is 0.5 approximately

More specifically, Fig. 6 shows how the proportion of working-age population changes over 1960–2050 in the five selected countries (i.e. Nepal, Philippines, Malaysia in Cluster A, China and Czech in Cluster B), to demonstrate the demographic window in a more detailed and intuitive way. If this proportion is higher 67% (i.e. when the total dependency ratio reaches 0.5), the demographic window is open. Among the three countries in Cluster A, the window in Philippines will not open before 2050, despite the increase in proportion of working-age population; currently, the proportion in Nepal is about 64%, and will reach 67% around 2025 when its demographic window appears; the proportion in Malaysia reaches its maximum level now, implying that the window has opened to the largest extent. Among the two countries in Cluster B, the demographic window in Czech has already closed, with the proportion of working-age population falling below 67% since 2015; in China, the proportion has been declining from its maximum level since 2010, signifying that its demographic window is going to close soon.

As shown Figs. 5 and 6, there is great heterogeneity in the pattern of demographic windows, indicating that some demographic interflow among the B&R countries for economic complementarity would be desirable. As suggested by Bloom and Canning (Bloom et al. 2003), these windows of opportunities can be turned into demographic dividends only if the labor force can be absorbed into the job market and transformed into gainful employment. This can only happen if the quality of the workforce is of high standard with good infrastructure in the society. Therefore, if the B&R strategy can increase employment opportunities through economic cooperation such as cross-border trade and investment, countries in Cluster A can be the destination for relocation of labor intensive industries and might also be able to enjoy the demographic dividend; meanwhile, countries in Cluster B can seize the chance to upgrade their industrial structure, to make use of immigration policies to import young talents from aboard, and to promote more interflow among the countries. Thus, a win–win outcome might be realized.

#### Wealth and well-being in the B&R region

**Fig. 7:**
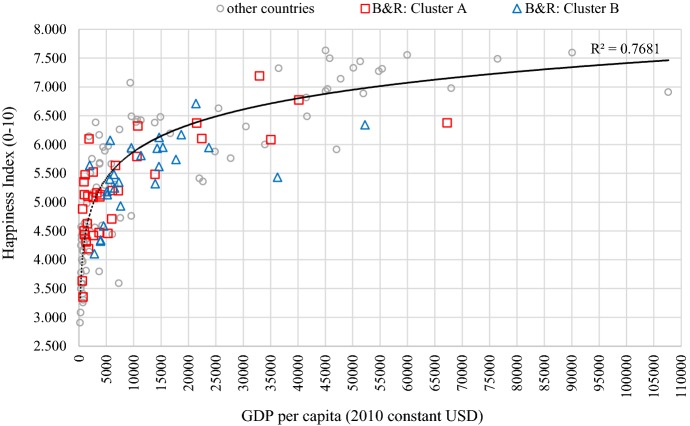
GDP per capita and the Happiness Index in 2015. Due to limitation of data, five countries (Brunei Darussalam, Oman, Syrian Arab Republic, Timor-Leste, and Maldives) are not included

**Fig. 8:**
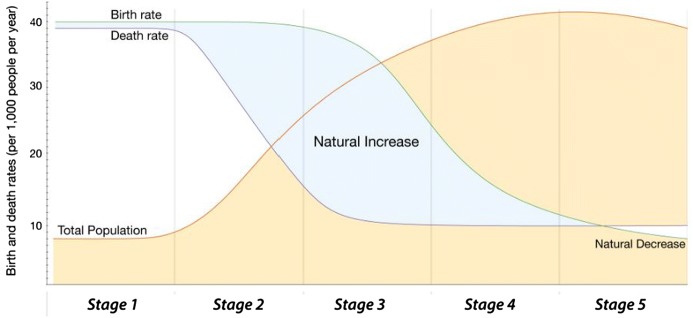
Five stages of the demographic transitions. Source: http://ourworldindata.org/data/population-growth-vital-statistics/world-population-growth

To explore whether the B&R initiative will lead to enhancement in wealth and well-being together, the relationship between the happiness index (as a measure of well-being) and GDP per capita (as a measure of wealth) is visualized. As shown in Fig. 7, the world trend line between the two indicators shows a curvilinear rather than a linear relationship: when the GDP per capita is at a low level, especially below 5000 USD, the two indicators are strongly related, meaning that a small increase of wealth is likely to cause a large improvement in the happiness index; when the GDP per capita is at a very high level, the increase of wealth seems to be less related with the happiness index, that is a large increase of GDP per capita may not be accompanied by a large increase of the happiness index. In Fig. 7, the distribution of Cluster A in the B&R region is more concentrated in low levels of wealth, especially about 60% of the countries are of GDP per capita less than 5000 USD. In contrast, the distribution of Cluster B is more dispersed, especially only about 20% of the countries in this cluster are of GDP per capita less than 5000 USD. These differences in the distribution between the two clusters imply that if the B&R strategy can succeed in stimulating economic development, the well-being of the people living in the countries of Cluster A will be greatly improved, probably by a larger magnitude than in the countries of Cluster B. However, it still needs to be emphasized that economic development should not be the only focus of the B&R strategy, that more attention should be paid to the well-being of the people within China and beyond its border, and that more efforts should be devoted to expending not only on the richness of economic development but also on the richness of human well-being in the B&R region.

## Conclusion and discussion

In this study, the demographic heterogeneities among the B&R countries have been identified. First, there are great variations in the current stages of demographic transitions across the B&R countries, 20% of the member countries are at the fifth stage and their population size has already started to decline, indicating the challenge of labor force shortage and a rapidly aging population, the rest of the member countries are at the third or fourth stages where the population grows at a moderate or low pace, providing a wider window of enjoying a population dividend of a smaller total dependency ratio.

Second, the present findings have identified that population aging is the common challenge where almost all the B&R countries must brace themselves by the middle of the twenty-first century. The era of “a super-aged society” will arrive earlier in the countries located in Eastern Europe, East and Southeast Asia, while later, in the countries located in the Middle and South Asia, and Africa. This difference in the timing of “aging” leaves much room for regional cooperation in terms of labor migration and technology exchanges, so that some of the B&R countries can learn from the experiences in those super-aged societies and find their own way to cope with population aging in the future. The very aged societies can also explore effective ways to maintain their economic growth by developing their silver hair market to respond to the changing consumption pattern of an aged society.

Third, the model-based cluster analysis has classified the 65 countries into two groups. Compared to Cluster A, countries in the Cluster B have much better socioeconomic positions, especially, they have higher GDP per capita, higher rate of tertiary school enrolment and female labor participation, and smaller gender gap in the labor market. Besides, the windows in the countries of Cluster B have already closed or are about to close very soon; in contrast, the demographic windows in the countries of Cluster A will open or remain open in the coming three decades.

As seen from the demographic heterogeneities in the B&R region, policies should be tailor-made to the countries’ population profiles and the people’s need. For Cluster B, the seriousness and inevitable aging situation calls for social and economic policies to create sufficient job opportunities in the labor market for the elderly population and provide a more friendly environment for them so that they can be empowered to contribute their accumulated human capita (including skills, knowledge, and experience) to society again, at the same time, appropriate immigration policies are need to attract and import the young talents from abroad to compensate for their manpower shortage in Cluster B. However, given the vast diversity in the region covered by the B&R initiative in terms of culture, religion, and social regimes, the cross-border movement of people might very sensitive to some extent, therefore, migration should be handled in a more careful way. For Cluster A, a different set of policies might be considered. As can be learnt from the western countries, having a higher proportion of young people can sometimes be problematic. A community with high youth unemployment can be a source of discontent. It calls for creation of new jobs to transfer the young labor force into productive employment so that the time-limited demographic opportunities can be turned into real dividends. However, education and training are essential to ensure quality workforce available to face the challenges. Gender equality is also another contentious issue in Cluster A countries which can be related to education opportunities for women and/or some gender cultural specific issues, for example, early or teen marriages among women in African and South Asian countries. Female labor potentials have not been developed and realized in most of the countries in Cluster A. However, if the policy fails to recognize potential human resources, if the policy fails to invest in equipping young talents, and if the policy fails to unleash the potentials of women labor force, the demographic dividend, oppositely could turn into a demographic burden (Leela Visaria 2017).

Fourth, the curvilinear relationship between GDP per capita and the happiness index has indicated that in less developed countries, a small increase in wealth might lead to a large improvement in well-being in that the basic needs of livelihood are being satisfied; whereas in more developed countries, to increase people’s well-being may not only call for economic growth but also improvement in social support, healthy life, freedom of making life choices, and so on. It has also been revealed that in the countries of Cluster A (with low GDP per capita), the stimulation of economic development through the B&R initiative is more likely to effectively enhance their well-being, and the well-being of the people living in this region should be included as the ultimate goal of the B&R strategy.

In the long run, in order to achieve mutual benefits and sustainable development through the B&R strategy, a “to know, comprehend, and embrace diversity” mindset is very needed for cross-regional collaboration. It is important to be able to co-create and co-sharing with possible collaborations and joint development in these countries. This should also be seen as a mutually beneficial venture and should not only be limited to economic development. However, none of the cooperation and bonding relationship can be materialized without deep understanding of one another. This study has unveiled and demonstrated that within the B&R region there exists much demographic heterogeneities, and such heterogeneities can serve as the foundation of socioeconomic cooperation. Hence, full respect should be yielded to one another’s differences, and appropriate policies should be employed to transform the exiting differences into competitive advantages for joint development.

## Appendix

See Fig. 8.
